# Editorial: New Paradigms in Neuroscience and Related Targets for Drug Discovery

**DOI:** 10.3389/fphar.2020.00750

**Published:** 2020-05-15

**Authors:** Salvatore Salomone

**Affiliations:** Department of Biomedical and Biotechnological Sciences, University of Catania, Catania, Italy

**Keywords:** drug discovery, animal model, neuropharmacology, neurodegeneration, brain ischemia, addiction, alzheimer's disease

This Research Topic received contributions through two fields: Frontiers in Pharmacology and Frontiers in Neuroscience. We would like to thank all the authors who made possible the success of this topic with their contributions, which give us a glimpse of how productive and diversified is the basic research in neuropharmacology, targeted to drug discovery.

A first group of papers focused on stroke, neuroprotection, and recovery. Until recently, many experimental paradigms have tested the neuroprotective effects of treatments carried before the ischemic insult. However, though helpful in elucidating pathophysiological mechanisms, these studies provided very little hints for human therapy, because stroke patients are commonly seen and treated after the occurrence of brain ischemia. Thus, therapeutic strategies aiming at improving recovery might realistically have more translational potential. A review (Balbinot and Schuch) examines neuromodulatory systems involved in stroke recovery before, during or after rehabilitation and propose them as targets for novel drug treatments. Recovery from ischemic stroke relies on neuronal plasticity; in particular, cortical and striatal cellular mechanisms underlying motor learning also affect post-stroke compensatory relearning. Another review (Malone et al.) examines immunomodulatory therapeutic approaches to reduce neurotoxicity and/or to promote neurorestoration and tissue repair. Drugs targeting innate immunity [e.g. biotechnological agents toward interleukin-1, tumor necrosis factor alpha (TNFa), etc … ], are likely to counteract neuronal injury in the acute phase, while drugs targeting the adaptive immune response (regulatory T and B cells), are more suitable to affect the repair processes, and might be used over a longer therapeutic window. Furthermore, the observation that ischemic stroke itself induces alterations in immunity, potentially responsible for post-stroke dysbiosis and gut-induced neuroinflammation, points to immunomodulatory therapeutic strategies to counteract mechanisms out of central nervous system (CNS), capable of impacting on stroke outcome. Two experimental papers propose novel potential targets for vascular-dependent brain disorders; one points to the adiponectin receptor, showing that adiponectin and an adiponectin receptor agonist exert neuroprotective effects against oxygen/glucose deprivation (Liu et al.), while the other points to endothelial progenitor cell-mediated angiogenesis after cerebral ischemia–reperfusion, a process stimulated by dichloroacetate (Zhao et al.). Obviously, both preclinical models need further validation, but at least they provide novel insights in the pathophysiology of brain ischemia.

Another group of papers focus on neurodegenerative diseases, particularly Alzheimer disease (AD), and neuroinflammation. Despite intense efforts to understand the cellular and molecular mechanisms leading to neurodegeneration, disease modifying drugs for AD are still unavailable. One paper points to the usefulness of current animal models of AD, particularly discussing the translational potential of transgenic mice and transgenic rats (Cuello et al.). The impact of drug treatments on cognition and memory relies on animal paradigms for drug testing, i.e. experimental models which provide functional (behavioral) data predictive of human outcomes. Clinical developing treatments for AD requires the identification of biomarkers to identify an ongoing AD process before clinical presentation, refine clinical trial design and set meaningful endpoints. One perspective paper (Hampel et al.) examines the potential of exploiting “liquid biopsies,” e.g. neural exosome proteins and/or miRNAs. The significance of circulating miRNAs in sporadic AD needs further clarification, which may not only provide novel biomarkers but also offer new miRNA-targeted therapies. Based on data suggesting that antidepressants reduce the risk to develop AD and may even exert neuroprotective effects in AD, an experimental research paper (Torrisi et al.) further explores the connection between AD and depression, testing the hypothesis that fluoxetine and vortioxetine may prevent memory deficits and depressive-like phenotype induced by intracerebroventricular injection of beta amyloid. The results indicate that fluoxetine and vortioxetine can prevent both cognitive deficits and depressive-like phenotype in this model, an effect that might be related to transforming growth factor β1 (TGF-β1). Improving cognitive functions, particularly those related to memory mechanisms, including long-term potentiation and long-term depression (LTD), is one of the approaches in drug discovery for neurodegenerative disorders. An original paper (Mango and Nistico) investigates the role of acid-sensing ion channel 1a in synaptic plasticity and demonstrates, in the LTD paradigm in mouse hippocampus, an interplay between them and glutamate *N*-methyl-d-aspartate receptors. These channels may become a therapeutic target for improving cognitive functions in neurodegenerative disorders. A review (Grassi et al.) examines the enzymes and receptors involved in sphingosine-1-phopshate production as potential drug target for different neurodegenerative diseases. Starting from the approved use of fingolimod in multiple sclerosis, the authors proceed to Alzheimer, Parkinson, and Huntington diseases. Besides some common mechanisms related to neuroinflammation, the authors consider specific genetic conditions in sphingolipid metabolism, where mutations of enzymes are responsible for pathological phenotypes, suggesting that they may serve as druggable targets. An original article examines the therapeutic potential of extracellular vesicles derived from mesenchymal stem cells in spinal cord injury (Lu et al.). The results are encouraging, showing an improvement of blood-spinal cord barrier, with reduction of its permeability, as well as a neural protection. The study of extracellular vesicles as therapeutic agents has seen growing efforts in recent years, not only for their potential in CNS diseases, but also for other organs and systems. The translation of such experimental data, however, requires not only validation in human studies, but also to address problems related to large scale production, batch to batch reproducibility, and standards of quality. A mini review examines the role of microglia in the defense against brain tumors, in particular glioblastoma multiforme (Prionisti et al.). The novel idea here is to target two-pore domain potassium channel expressed by microglia to modulate its motility (production of processes) and its release of cytokines, particularly IL-1beta.

Two papers regard epilepsy. One is a review on the potential repurposing of bumetanide, a loop diuretic, related to its interaction with sodium–potassium–chloride (Na–K–Cl) cotransporters in neurons (Kharod et al.). Bumetanide may lower cytosolic Cl^−^ which facilitate GABA-induced hyperpolarization; based on this grounds, bumetanide has been studied in both preclinical and clinical settings, and seems promising for temporal lobe epilepsy, autism, and schizophrenia. The other paper is an original article testing the hypothesis that tribbles pseudokinase 3 (TRIB3) plays a key role in seizures and neuronal apoptosis (Zhang et al.). By using a kainic acid rat seizure model, the authors were able to demonstrate that TRIB3 upregulation inhibits AKT and induces neuronal apoptosis. They conclude that TRIB3 may represent a potential pharmacological target for the treatment of epilepsy.

A group of papers deal with addiction and addiction-related substances (cannabinoids, opiates) which hold therapeutic potential. One review focus on the role of the insula in addiction (Ibrahim et al.). Insula is a region of cerebral cortex recently implicated in several critical mechanisms of addiction. Originally studied for nicotine addiction, for which more data are available, recently insula has been also involved in other substance use disorders, including alcohol, opiates, cannabis. The authors not only review the role of insula, but also examine and discuss a recent technique, transcranial magnetic stimulation, which may offer potential benefits in addiction treatment. Two original articles provide novel insights into the therapeutic potential of cannabinoids. One paper examines the effect of systemic or intra-hippocampal administration of cannabidiol on hyperactivity shown by Gria1−/−, a mouse line deficient in AMPA-type glutamate receptor GluA1 subunit, and found that it is effective in dampening the activity of hyper excitable hippocampus (Aitta-Aho et al.). The role of selective inhibition of the dorsal hippocampal principal neurons in Gria1−/− mice was confirmed by inhibitory designer receptors activated by designer drug (DREADD). This paper suggests that cannabidiol holds potential for treating syndromes with hyper excitable hippocampus. The other paper explores the impact of pharmacological inhibition of the fatty acid amide hydrolase (FAAH), which increases endogenous levels of arachidonylethanolamide (anandamide), on cognitive functions. The authors provide evidence of attention enhancement in adolescent mice following FAAH inhibition (Contarini et al.). This finding supports further studies on FAAH inhibitors in human conditions, such attention-deficit/hyperactivity disorder (ADHD), where cannabis use as automedication provides some relief, but produces also adverse reactions. A brief research report examines the effects of non-competitive (receptor-inactivating) antagonists of kappa opioid receptors (Chavkin et al.). The activation of these receptors has been related to anxiogenic, dysphoric, and cognitive disrupting effects of repeated stress, suggesting that their antagonists could be used for treating some stress-related disorders. Norbinaltorphimine produces cumulative kappa opioid receptor inactivation, at doses much lower of those required for achieving acute effective antagonism. Such a mechanism of cumulative inactivation may improve safety, selectivity, and clinical efficacy of kappa antagonism, which may be exploited for treating stress-related conditions, including anxiety, depression, and addiction.

An interesting paradigm shift in drug discovery for CNS drugs is illustrated in a theoretical paper (Geerts and Barrett), proposing a computer model of biological processes, informed by preclinical and clinical data, which could be used as a tool to integrate a large amount of data to address key points in pharmacodynamics (PD), pharmacokinetics (PK), and PD–PK interactions and profiles. This kind of platform, named Quantitative Systems Pharmacology (QSP), aims at modeling, *in silico*, brain circuitry relevant for specific diseases and/or symptoms, to improve the success rate of CNS drug discovery programs, reduce cost and animal use, increase the speed of the processes. While this paper deals with the general paradigm of CNS drug discovery, two others examine the need of appropriate models for specific neuropsychiatric conditions. One specifically examines the conditions that should be fulfilled by preclinical models of post-traumatic stress disorder (PTSD), which should take into account and try to reproduce in the animal model the symptoms as classified for humans in Diagnostic and Statistical Manual of Mental Disorders, Fifth Edition (DSM-V) (Torrisi et al.). Interestingly, the currently available criterions for human PTSD are consistent with impairment of dopaminergic mechanisms and points to potential dopaminergic-based pharmacotherapies for PTSD to address a yet unmet medical need. Another paper deals with a preclinical model of anorexia nervosa, the activity-based anorexia, where rodents are tested for their preference for feeding over performing exercise (Hurel et al.). This study reports sex-related differences in the preference for exercise over feeding in fed animals, but sex-independent preference for feeding in food-restricted animals. The authors conclude that this model is not adequate to discriminate and quantify running and feeding drives, which are critically affected in anorexia, and should be considered when testing experimental drug treatments.

In conclusion, by pointing to diverse areas of research in neurophysiology and neuropharmacology, from vascular and degenerative diseases to neuropsychiatric conditions, involving different neurotransmitters and neuromodulators, this Research Topic indicates that several new paradigms are available and, at least some of them, may help in defining novel druggable targets. Worthy of note, several studies propose novel preclinical (animal) models and/or critically re-examine the available ones. The validation of preclinical models is an absolute requirement to effectively test experimental hypothesis that may allow us to translate novel drug treatments in clinical settings.

## Author Contributions

SS is the sole author of this editorial. He conceived and wrote this manuscript.

## Conflict of Interest

The author declares that the research was conducted in the absence of any commercial or financial relationships that could be construed as a potential conflict of interest

